# Modified Naples prognostic score for evaluating the prognosis of patients with obstructive colorectal cancer

**DOI:** 10.1186/s12885-023-11435-8

**Published:** 2023-10-05

**Authors:** Junnan Gu, Shenghe Deng, Zhenxing Jiang, Fuwei Mao, Yifan Xue, Le Qin, Jianguo Shi, Jia Yang, Huili Li, Jie Yu, Ke Liu, Ke Wu, Yinghao Cao, Kailin Cai

**Affiliations:** 1 Department of Gastrointestinal Surgery, Union Hospital, Tongji Medical College, Huazhong University of Science and Technology, Wuhan, 430022 Hubei China; 2 Cancer Center, Union Hospital, Tongji Medical College, Huazhong University of Science and Technology, Wuhan, 430022 Hubei China; 3 Department of Gastrointestinal Surgery, The Central Hospital of Wuhan, Tongji Medical College, Huazhong University of Science and Technology, Wuhan, 430022 Hubei China; 4 Department of Colorectal Anal Surgery, Jingzhou Central Hospital, The Second Clinical Medical College, Yangtze University, No. 60 Jingzhong Road, Jingzhou, 434020 Hubei Province China

**Keywords:** Obstructive colorectal cancer, Modified Naples prognostic score, Prognostic factors, Inflammatory, Nutritional status

## Abstract

**Background:**

Inflammatory, immune, and nutritional status are key factors in obstructive colorectal cancer (OCRC). This study aims to investigate the value of modified Naples prognostic score (M-NPS) in evaluating OCRC prognosis.

**Methods:**

A total of 196 OCRC patients were retrospectively analyzed to construct M-NPS based on serum albumin (ALB), total cholesterol (CHOL), neutrophil:lymphocyte ratio (NLR), and lymphocyte:monocyte ratio (LMR), and then they were divided into three groups. The Kaplan–Meier (KM) method and Cox proportional hazard regression analysis were performed for overall survival (OS) and disease-free survival (DFS) of OCRC patients.

**Results:**

Patients with high M-NPS had worse OS and DFS (*P* = 0.0001, *P* = 0.0011). Multivariate COX analysis showed that M-NPS was an independent prognostic factor for OCRC patients. Patients in the M-NPS 2 group had significantly worse OS (hazard ratio [HR] = 4.930 (95% confidence interval [95% CI], 2.217–10.964), *P* < 0.001) and DFS (HR = 3.508 (95% CI, 1.691–7.277), *P* < 0.001) than those in the 0 group.

**Conclusion:**

M-NPS was an independent prognostic factor for OCRC patients; it might provide a potential reference for immunonutritional intervention in patients with obstruction.

## Background

Colorectal cancer (CRC) is the third most prevalent cancer in the world, with acute colonic obstruction present in 8–29% of CRC patients [1–3]. Patients with obstructive CRC (OCRC) are often associated with poor nutritional status, prognosis and quality of survival. In addition to surgery as a routine emergency management, the increase in self-expanding metal stent (SEMS) implantation offers a new option to relieve obstructive symptoms [1, 2]. However, the overall prognosis of OCRC patients is still not promising. The construction of new prognostic biomarkers could better assist clinicians in making rational treatment decisions.

The inflammatory, immune and nutritional status of patients are considered to be closely related to the prognosis of CRC [4–7]. Inflammation can significantly increase the risk of cancer development, whereas OCRC usually has more serious local and systemic inflammatory reactions [8, 9]. The CRC tumor microenvironment has complex immune responses, and immunotherapy has become an important pillar of CRC treatment in recent years; moreover, different immune scoring systems for assessing CRC prognosis are constantly being developed [6]. In addition, the relationship between nutritional status and prognosis of CRC receives increased attention, especially in OCRC patients, where nutritional status significantly affects postoperative recovery, prognosis, and quality of survival [7, 10]. Therefore, many inflammatory immune markers have been studied to evaluate the prognosis of CRC. Neutrophil:lymphocyte ratio (NLR), platelet:lymphocyte ratio (PLR), lymphocyte:monocyte ratio (LMR), systemic immune–inflammatory index (SII), prognostic nutrition index (PNI), and Naples prognostic score (NPS) were found to be independent prognostic factors of CRC [7, 11–16]. However, few biomarkers used to evaluate the prognosis of OCRC patients require further exploration.

NPS is a prognostic score constructed by Galizia Gennaro et al. based on serum albumin (ALB), total cholesterol (CHOL), NLR, and LMR [15]. The value of NPS in predicting the prognosis of CRC, metastatic CRC (mCRC), gastric cancer, duodenal ampullary cancer, and non-small cell lung cancer [15, 17–20] has been confirmed by relevant studies, but no relevant exploration in OCRC has been conducted. In this study, we established a modified NPS (M-NPS) based on a cohort of patients with OCRC and explored its value in assessing the prognosis of OCRC.

## Methods

### Patient cohort

**Fig. 1:**
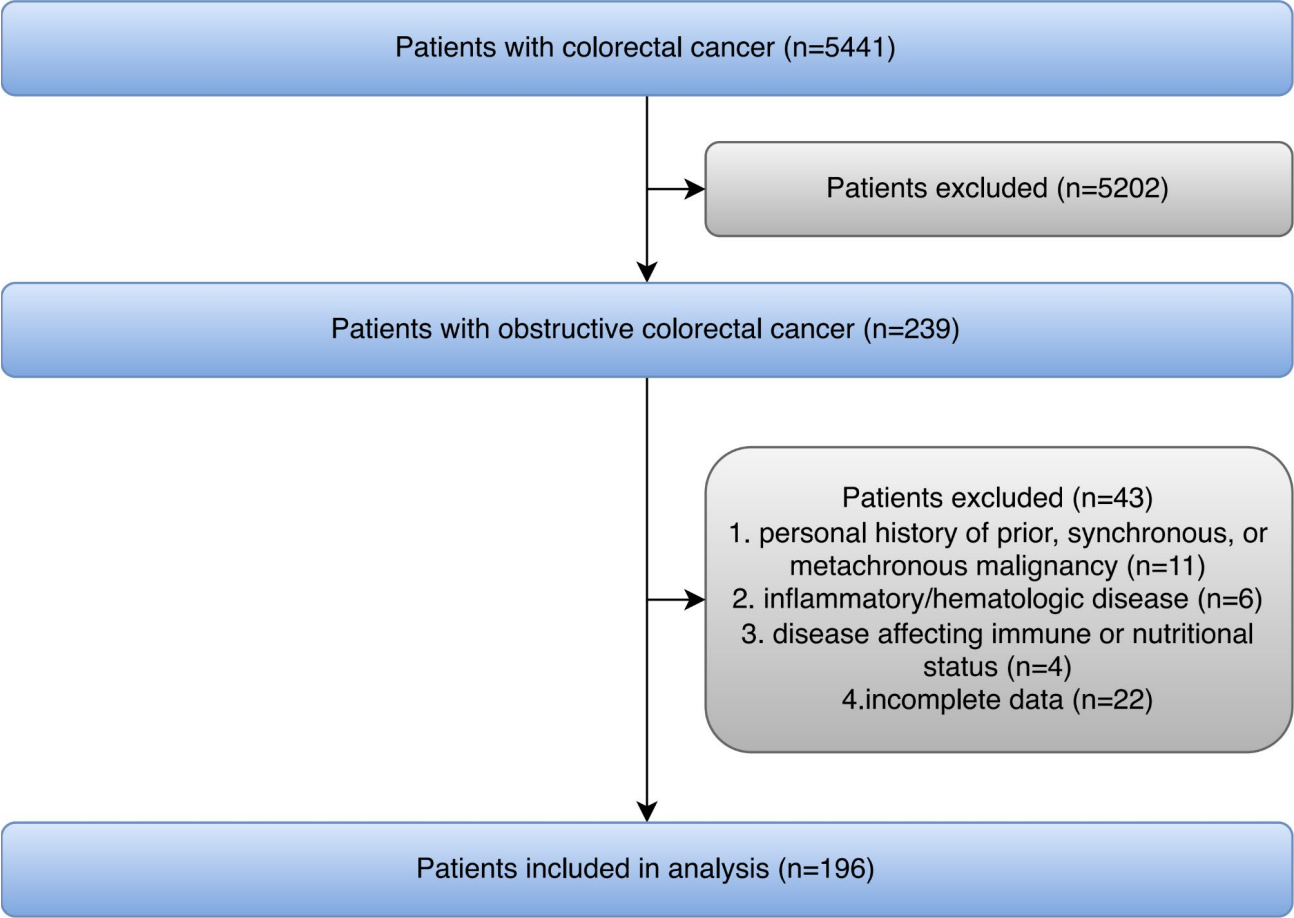
Flow diagram of study population selected

This study finally included 196 OCRC patients, who received treatment at Wuhan Union Hospital, Wuhan Central Hospital, and Jingzhou Central Hospital from December 2014 to May 2018 (Fig. 1). All patients with CRC complicated by colorectal obstruction confirmed by computerized tomography (CT), magnetic resonance imaging (MRI), and endoscopy were eligible. The exclusion criteria were as follows: personal history of prior, synchronous, or metachronous malignancy; inflammatory/hematologic disease and disease affecting immune or nutritional status. The patients were generally followed up every three months by the outpatient service with CT in the first two years and then annually for the next three to five years when no evidence of recurrence was observed. For the patients who did not visit our hospital as scheduled, telephone interviews were conducted to obtain treatment information and progression status. The end of follow up was in September 2021. Median follow-up time was 39 months. The present study has been approved by the ethics committee of Wuhan Union Hospital (No.2018-S377) and conducted in accordance with the ethical standards of the World Medical Association Declaration of Helsinki.

### Procedures of treatment

All the enrolled patients received resection of colorectal cancer, and some patients received preoperative SEMS implantation according to their admission conditions and indications. 41 patients were treated with SEMS implantation and underwent surgery within two weeks after SEMS implantation. The interval between bridging is approximately 7–14 days. 104 patients underwent laparoscopic resection of colorectal cancer, and 92 patients underwent open resection of colorectal cancer. Five of the enrolled patients had distant metastases (liver metastases), all of which were single, and radical resection of the metastatic lesion was performed.

### Data collection

The following clinicopathological features were collected from the patient’s medical records: age, sex, body mass index (BMI), American Society of Anesthesiologists Physical Status Classification (ASA class), tumor location, tumor size, tumor differentiation, TNM stage [21], vascular tumor thrombus, nerve invasion, SEMS implantation, chemotherapy, radiotherapy, lymph node ratio (LNR), laboratory examination included ALB, CHOL, whole blood count (neutrophils, lymphocytes, and monocytes), and CEA. Blood samples were taken within two weeks prior to radical surgery or stent placement, and M-NPS was calculated based on the results of this blood sample.

### Construction of M-NPS

**Fig. 2:**
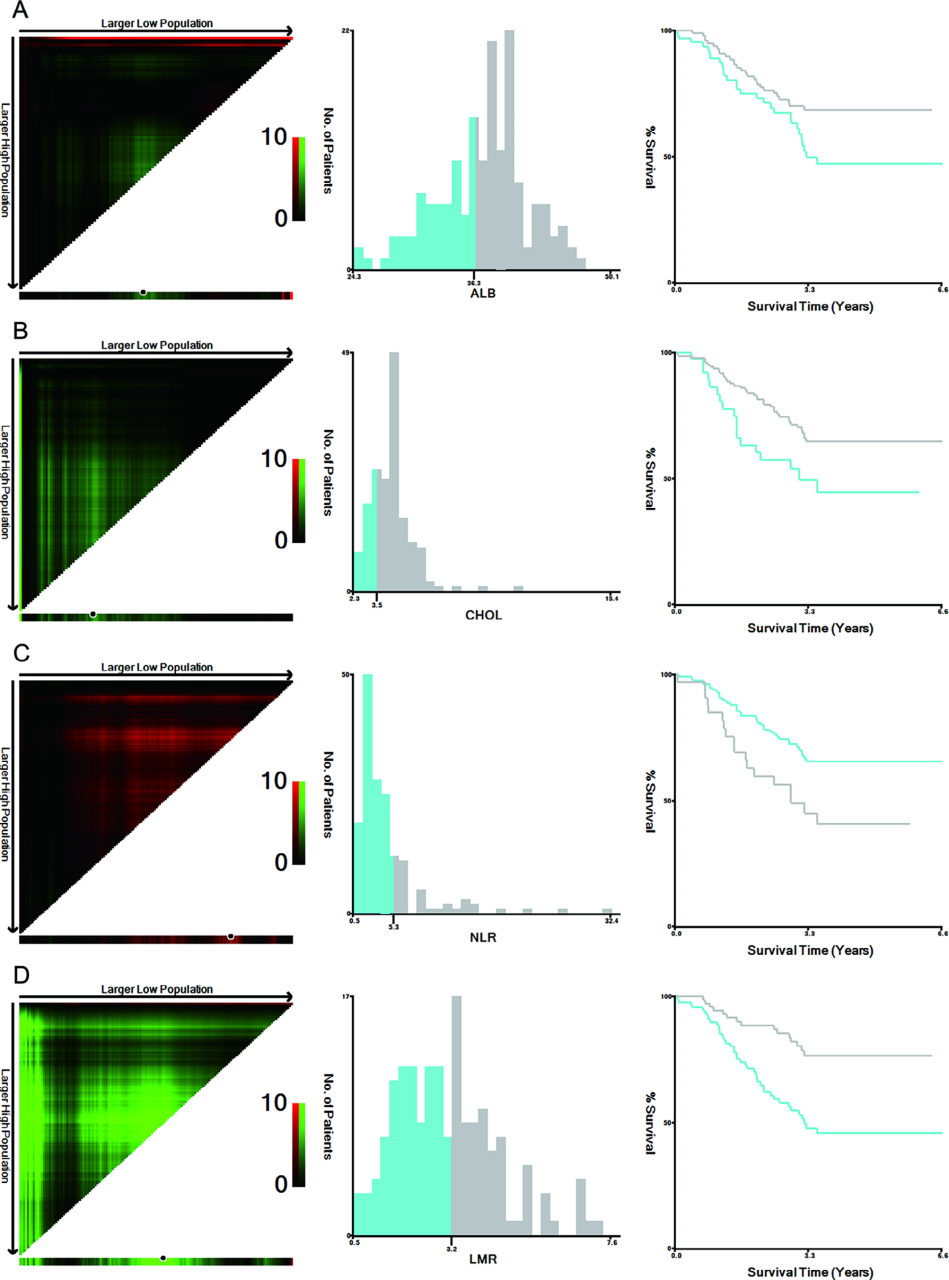
Optimal cut-points of ALB, CHOL, NLR, and LMR were determined by X-tile analysis. The plot shows the χ2 log-rank values produced when dividing the cohort with optimal cut-points, producing high and low population. X-tile plots are shown in the left panels, red coloration of cut-points indicates an inverse correlation with survival, whereas green coloration represents direct associations. The optimal cut-point highlighted by the black circle in the left panels is shown on a histogram (middle panels), and Kaplan–Meier plots are presented in right panels. In terms of overall survival, the optimal cut-points of ALB **(A)**, CHOL **(B)**, NLR **(C)** and LMR **(D)** were 36.3, 3.48, 5.33, and 3.19, respectively

NPS was composed of ALB, CHOL, NLR, and LMR. Since conventional NPS did not demonstrate significant evaluation significance in this study cohort, based on the particularity of patients with obstruction, x-Tiles 3.6.1 (Yale University, New Haven, CT, USA) was used for data analysis to obtain the optimal cut-points of the four indicators (Fig. 2) [22]. The modified prognostic score composition is as follows: the score of ALB concentration > 36.3 g/L was 0, ≤ 36.3 g/L was 1; CHOL concentration > 3.48 mmol/L was 0, ≤ 3.48 mmol/L was 1; NLR ≤ 5.33 was 0, > 5.33 was 1; LMR > 3.19 was 0, and ≤ 3.19 was 1. The scores of the four indicators were M-NPS, and the patients with M-NPS of 0, 1 and 2, and 3 and 4 were assigned to groups 0, 1, and 2, respectively, for analysis.

### Statistical analysis

The main result of this study is the overall survival (OS), and the secondary result is disease-free survival (DFS). OS was defined as the time from the diagnosis of CRC with obstruction to the date of death, follow-up, or end of follow-up, whichever came first. DFS was calculated as the interval between the diagnosis of CRC with obstruction and the first documentation of disease recurrence, death, or end of follow-up, whichever came first. Continuous variables were expressed as the mean ± standard deviation or median and interquartile ranges (IQR). Mann–Whitney U or Kruskal–Wallis Chi-Squared was used to compare the differences of variables between groups. The categorical variables were expressed as frequency and percentage, and the differences between groups were compared using Pearson Chi-square test, Fischer precision test, or Spearman’s rank correlation test. Some variables were dichotomized using normal or median values, and X-tile software was used to analyze the four basic indicators of M-NPS for classification conversion. Kaplan–Meier (KM) method and log-rank test were applied to compare the survival difference between groups. The receiver operating characteristic (ROC) curve of 12-month, 24-month, and 36-month was established, and the area under curve (AUC) was analyzed to compare the predictive ability of the prognosis scoring system. Cox proportional hazard regression was used to calculate hazard ratios (HRs), and the corresponding 95% confidence intervals (95%CIs). Variables with P < 0.05 in univariate analysis were included in the multivariate analysis. Statistical analyses were performed using IBM SPSS Statistics 26.0 (IBM Corp., Armonk, NY, USA) and R 4.2.1 (R Foundation for Statistical Computing, Vienna, Austria). All analyses were two-sided, and P values < 0.05 were considered statistically significant.

## Results

### Patient characteristics and correlation between M-NPS and patients’ clinicopathological characteristics

A total of 196 OCRC patients were included in the final statistical analysis (Fig. 1). The median age of the patients was 65 years, with 102 males and 94 females. Median OS and DFS were 39 and 36 months, respectively (Table 1). According to M-NPS, patients were divided into three groups: 57 cases in group 0, 110 cases in group 1, and 29 cases in group 2. M-NPS showed significant inter-group differences in age (*P* = 0.005), sex (*P* = 0.010), ASA class (*P* = 0.011), tumor size (*P* = 0.047) and four M-NPS factors (*P* < 0.001), whereas no significant inter-group differences were found in other variables (Table 1).

**Table 1:** Relationships between Modified Naples prognostic score and clinicopathological characteristics

		Modified Naples prognostic score			
Variables	Total	Group 0	Group 1	Group 2	P*
**Age(yr)**	65(55–71)	63 (54–67)	65(55–71)	71(64–79)	0.005
**Sex**					0.010
Male	102(52.0)	21(36.8)	61(55.5)	20(69.0)	
Female	94(48.0)	36(63.2)	49(44.5)	9(31.0)	
**BMI (mean ± SD)**	22.1 ± 2.8	21.9 ± 2.7	22.2 ± 2.7	22.0 ± 3.2	0.768
**ASA class**					0.011
1	5(2.5)	2(3.5)	3(2.7)	0(0.0)	
2	126(64.3)	42(73.7)	68(61.8)	16(55.2)	
3	35(17.9)	10(17.5)	19(17.3)	6(20.7)	
4	30(15.3)	3(5.3)	20(18.2)	7(24.1)	
1/2	131(66.8)	44(77.2)	71(64.5)	16(55.2)	0.090
3/4	65(33.2)	13(22.8)	39(35.5)	13(44.8)	
**Location**					0.214
Right colon	63(32.1)	19(33.3)	38(34.5)	6(20.7)	
Left colon	78(39.8)	19(33.3)	42(38.2)	17(58.6)	
Rectum	55(28.1)	19(33.3)	30(27.3)	6(20.7)	
**Size (cm)**					0.047
d<5	97(49.5)	36(63.2)	49(44.5)	12(41.4)	
d ≥ 5	99(50.5)	21(36.8)	61(55.5)	17(58.6)	
**Differentiation**					0.487
Low	28(14.3)	5(8.8)	18(16.4)	5(17.2)	
Medium	136(69.4)	43(75.4)	74(67.3)	19(65.5)	
High	32(16.3)	9(15.8)	18(16.4)	5(17.2)	
**TNM stage**					
I	5(2.5)	1(1.8)	4(3.6)	0(0.0)	0.745
II	85(43.4)	26(45.6)	46(41.8)	13(44.8)	
III	94(48.0)	27(47.4)	53(48.2)	14(48.3)	
IV	12(6.1)	3(5.3)	7(6.4)	2(6.9)	
I/II	90(45.9)	27(47.4)	50(44.5)	13(44.8)	0.965
III/IV	106(54.1)	30(52.6)	60(55.5)	16(55.2)	
**Tumor**					
T1	6(3.1)	0(0.0)	6(5.5)	0(0.0)	0.237
T2	23(11.7)	5(8.8)	11(10.0)	7(24.1)	
T3	89(45.4)	30(52.6)	45(40.9)	14(48.3)	
T4	78(39.8)	22(38.6)	48(43.6)	8(27.6)	
T1/2	29(14.8)	5(8.8)	17(15.5)	7(24.1)	0.158
T3/4	167(85.2)	52(91.2)	93(84.5)	22(75.9)	
**Node**					0.675
N0	96(49.0)	27(47.4)	55(50.0)	14(48.3)	
N1	68(34.7)	18(31.6)	40(36.4)	10(34.5)	
N2	32(16.3)	12(21.1)	15(13.6)	5(17.2)	
N0^a^	96(49.0)	27(47.4)	55(50.0)	14(48.3)	0.946
N1/2	100(51.0)	30(52.6)	55(50.0)	15(51.7)	
**Metastasis**					1.000
M0	191(97.4)	56(98.2)	107(97.3)	28(96.6)	
M1	5(2.6)	1(1.8)	3(2.7)	1(3.4)	
**Vascular Tumor Thrombus**					0.195
No	150(76.5)	39(68.4)	89(80.9)	22(75.9)	
Yes	46(23.5)	18(31.6)	21(19.1)	7(24.1)	
**Nerve Invasion**					0.453
No	137(69.9)	40(70.2)	74(67.3)	23(79.3)	
Yes	59(30.1)	17(29.8)	36(32.7)	6(20.7)	
**LNR (%)**	2.6(0.0-14.4)	3.3(0.0-17.6)	0.0(0.0-14.2)	3.1(0.0-12.5)	0.786
**SEMS implantation**					0.068
No	155(79.1)	51(89.5)	83(75.5)	21(72.4)	
Yes	41(20.9)	6(10.5)	27(24.5)	8(27.6)	
**Chemotherapy**					0.303
No	110(56.1)	28(49.1)	67(60.9)	15(51.7)	
Yes	86(43.9)	29(50.9)	43(39.1)	14(48.3)	
**Radiotherapy**					0.353
No	190(96.9)	56(98.2)	107(97.3)	27(93.1)	
Yes	6(3.1)	1(1.8)	3(2.7)	2(6.9)	
**CEA (ng/ml)**	5.7(2.7–16.4)	5.1(2.4–12.4)	6.7(2.9–20.6)	5.5(3.5–17.6)	0.364
**NPS factors**					
ALB (g/L)	38.1(34.7–40.3)	39.8(38.3–42.6)	37.8(34.2–40.3)	33.6(31.3–35.9)	< 0.001
CHOL (mmol/L)	4.3(3.6–4.8)	4.4(4.3–4.8)	4.3(3.6-5.0)	3.0(2.6–3.3)	< 0.001
NLR	3.1(2.0-5.2)	2.0(1.6–2.5)	3.6(2.4–5.3)	6.2(4.3–13.6)	< 0.001
LMR	2.9(2.0–4.0)	4.4(3.6-5.0)	2.6(2.0-3.3)	1.8(1.3–2.2)	< 0.001

**ASA class adopted binary classification (1/2 vs. 3/4)**

**TNM stage adopted binary classification (I/II vs. III/IV).**

**Tumor stage adopted binary classification (T1/2 vs. T3/4)**

**Node stage adopted binary classification (N0 vs. N1/2)**

**Right colon: the proximal two-thirds of the transverse colon, ascending colon and caecum**

**left colon: the distal third of the transverse colon, splenic flexure, descending colon, sigmoid colon**

*P **was calculated by the Kruskal–Wallis tests and the analysis of variance (ANOVA) for continuous variables and the Chi-square test and the Spearman’s rank correlation test for categorical variables**

### Prognosis (OS and DFS) of patients based on M-NPS

**Fig. 3:**
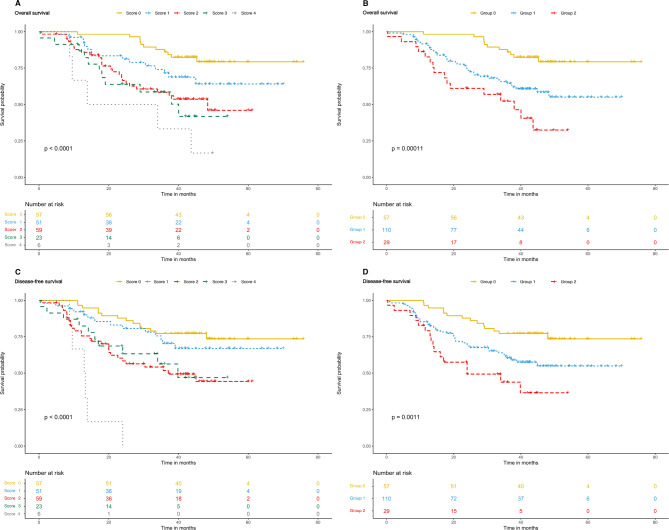
Kaplan–Meier survival analysis according to Modified Naples Prognostic Score and group. **(A)** Significant differences in the overall survival among patients were found with five scores (*P* < 0.0001). **(B)** Significant differences in the overall survival among patients were found with three score groups (*P* = 0.0001). **(C)** Significant differences in the disease-free survival among patients were found with five scores (*P* < 0.0001). **(D)** Significant differences in the disease-free survival among patients were found with three score groups (*P* = 0.0011)

The correlation between MNPS (0–4 scores and 0–2 groups) and OS/DFS were shown in Fig. 3. The KM method showed that MNPS (0–4 scores) were significantly associated with OS and DFS in OCRC patients (*P* < 0.0001, *P* < 0.0001). In the M-NPS group, the mean OS values of group 0,1, and 2 were 45.4, 32.2, and 27.2 months, respectively, and the mean DFS values were 42.7, 30.1, and 23.8 months. The OS and DFS in group 0 were significantly higher than those in groups 1 and 2 (*P* = 0.0001, *P* = 0.0011).

### Univariate and multivariate analysis of OCRC prognosis

The univariate and multivariate COX proportional hazard regression analysis of OS is shown in Table 2. The Univariate analysis showed that the M-NPS group was significantly correlated with OS (*P* = 0.003, *P* < 0.001). Further multivariate analysis confirmed that the M-NPS group was an independent prognostic factor of OS. Group 0 had significantly better prognosis than group 1 (HR = 2.635 (95%CI, 1.330–5.221), *P* = 0.006) and group 2 (HR = 4.930 (95%CI, 2.217–10.964), *P* < 0.001). Other independent prognostic factors for OS included TNM stage (*P* = 0.009) and nerve invasion (*P* = 0.016).

**Table 2:** Univariate and multivariate analyses of Prognostic factor for overall survival

	Univariate analysis		Multivariate analysis	
Variables	HR (95% CI)	P*	HR (95% CI)	P*
**Age**	1.022 (1.000-1.044)	0.048	1.008 (0.984–1.033)	0.516
**Sex**				
Male	1			
Female	0.916 (0.567–1.479)	0.720		
**BMI**	0.971 (0.890–1.058)	0.499		
**ASA class**				
1/2	1		1	
3/4	1.810 (1.115–2.938)	0.016	1.713 (0.950–3.092)	0.074
**Location**				
Right colon	1			
Left colon	0.756 (0.429–1.332)	0.333		
Rectum	0.852 (0.467–1.556)	0.602		
**Size**				
d<5	1			
d ≥ 5	0.986 (0.610–1.592)	0.954		
**Differentiation**				
Low	1			
Medium	0.838 (0.410–1.716)	0.630		
High	1.000 (0.427–2.342)	0.999		
**TNM stage**				
TNM I/II	1		1	
TNM III/IV	2.093 (1.255–3.490)	0.005	4.320 (1.446–12.903)	0.009
**Tumor**				
T1/2	1			
T3/4	1.980 (0.856–4.582)	0.110		
**Node**				
N0	1		1	
N1/2	1.753 (1.071–2.868)	0.026	0.488 (0.169–1.408)	0.185
**Metastasis**				
0	1			
1	1.882 (0.591–5.992)	0.285		
**Vascular Tumor Thrombus**				
No	1			
Yes	1.503 (0.875–2.582)	0.140		
**Nerve Invasion**				
No	1		1	
Yes	1.856 (1.126–3.059)	0.015	1.890 (1.124–3.178)	0.016
**LNR (%)**	2.096 (0.680–6.464)	0.198		
**SEMS implantation**				
No	1			
Yes	1.230 (0.681–2.222)	0.492		
**Chemotherapy**				
No	1			
Yes	0.961 (0.591–1.562)	0.872		
**Radiotherapy**				
No	1			
Yes	0.519 (0.072–3.742)	0.515		
**CEA**	1.000 (1.000–1.000)	0.132		
**M-NPS Group**				
0	1		1	
1	2.730 (1.398–5.332)	0.003	2.635 (1.330–5.221)	0.006
2	4.893 (2.259–10.601)	< 0.001	4.930 (2.217–10.964)	< 0.001

* (OS) Multivariate analysis adjusted for age, ASA class, TNM stage, node and nerve Invasion. We use a stepwise regression approach for multivariate analysis

At the same time, univariate and multivariate COX proportional hazard regression analysis was conducted for DFS (Table 3). The Univariate analysis showed that M-NPS group was significantly correlated with DFS (*P* = 0.015, *P* < 0.001). Further multivariate analysis confirmed that the M-NPS group was an independent prognostic factor of DFS. Group 0 had significantly better prognosis than group 1 (HR = 2.031 (95%CI, 1.104–3.737), *P* = 0.023) and group 2 (HR = 3.508 (95%CI, 1.691–7.277), *P* < 0.001).

**Table 3:** Univariate and multivariate analyses of prognostic factor for disease-free survival

	Univariate analysis		Multivariate analysis	
Variables	HR (95% CI)	P*	HR (95% CI)	P*
**Age**	1.012 (0.992–1.032)	0.236		
**Sex**				
Male	1			
Female	1.145 (0.721–1.820)	0.565		
**BMI**	1.021 (0.938–1.110)	0.636		
**ASA class**				
1/2	1		1	
3/4	1.742 (1.092–2.780)	0.020	1.763 (1.090–2.851)	0.021
**Location**				
Right colon	1			
Left colon	0.768 (0.446–1.323)	0.341		
Rectum	0.859 (0.480–1.539)	0.610		
**Size**				
d<5	1			
d ≥ 5	0.923 (0.581–1.465)	0.733		
**Differentiation**				
Low	1			
Medium	0.983 (0.483–2.001)	0.961		
High	1.256 (0.543–2.905)	0.594		
**TNM stage**				
TNM I/II	1		1	
TNM III/IV	1.655 (1.029–2.664)	0.038	1.902 (1.165–3.104)	0.010
**Tumor**				
T1/2	1			
T3/4	1.633 (0.783–3.407)	0.191		
**Node**				
N0	1			
N1/2	1.298 (0.815–2.066)	0.272		
**Metastasis**				
0	1			
1	1.851 (0.582–5.886)	0.297		
**Vascular Tumor Thrombus**				
No	1			
Yes	1.243 (0.721–2.145)	0.434		
**Nerve Invasion**				
No	1			
Yes	1.573 (0.964–2.567)	0.070		
**LNR (%)**	1.360 (0.422–4.388)	0.607		
**SEMS implantation**				
No	1			
Yes	1.163 (0.657–2.057)	0.604		
**Chemotherapy**				
No	1			
Yes	0.937 (0.586–1.496)	0.784		
**Radiotherapy**				
No	1			
Yes	1.133 (0.278–4.624)	0.862		
**CEA**	1.000 (1.000–1.000)	0.193		
**M-NPS Group**				
0	1		1	
1	2.128 (1.160–3.905)	0.015	2.031 (1.104–3.737)	0.023
2	3.691 (1.791–7.608)	< 0.001	3.508 (1.691–7.277)	< 0.001

* (DFS) Multivariate analysis adjusted for ASA class and TNM stage

**Fig. 4:**
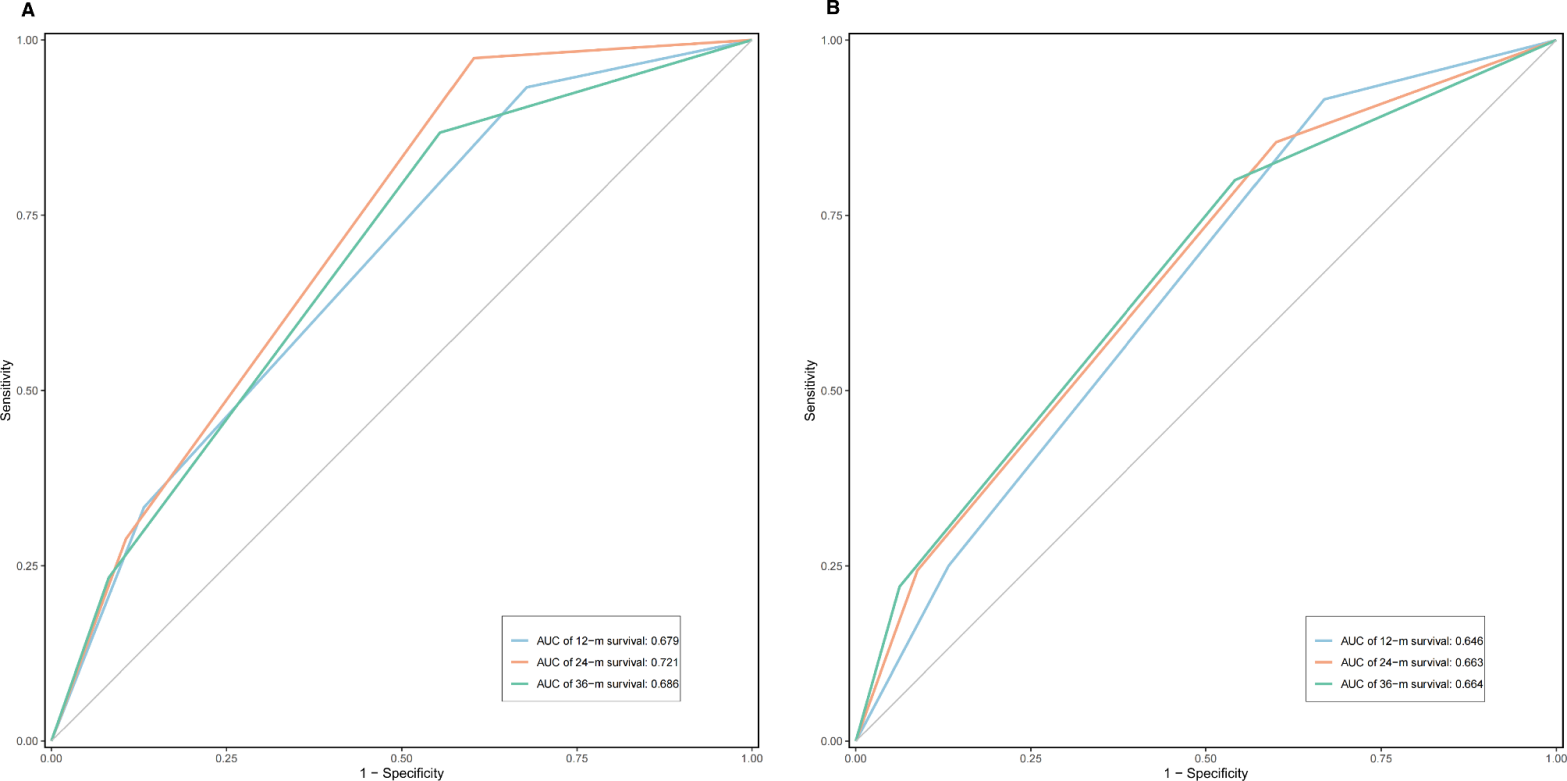
Time-dependent receiver operating characteristic (ROC) to assess Modified Naples Prognostic Score. Time-dependent ROC to assess the accuracy of Modified Naples Prognostic Score group in predicting overall survival **(A)** and disease-free survival **(B)** at 12, 24, and 36 months. **(A)** The area under the curve (AUC) values for 12-, 24-, and 36-month overall survival were 0.679, 0.721, and 0.686, respectively. **(B)** AUC values for 12-, 24-, and 36-month disease-free survival were 0.646, 0.663, and 0.664, respectively

Subsequently, the ROC curve was applied to compare the prediction ability of OS and DFS in the M-NPS group at 12, 24, and 36 months (Fig. 4). The results showed that the M-NPS group could effectively predict OS (AUC = 0.679, 0.721, 0.686) and DFS (AUC = 0.646, 0.663, 0.664) in OCRC patients within 36 months.

## Discussion

Although previous studies have explored the value of inflammatory or immunonutritional status indicators in evaluating the prognosis of OCRC [23–25], no study has combined these indicators to achieve a more effective prognosis assessment of patients with obstruction. A comprehensive score combined with these status indicators could provide a more comprehensive, effective, and intuitive reference basis for prognosis evaluation of OCRC patients, considering the complex inflammatory, immune, and nutritional status of OCRC patients. In this study, M-NPS composed of inflammatory, nutritional, and immune status-related indicators was constructed and validated as an independent prognostic factor for OS and DFS in OCRC. In addition, M-NPS demonstrated good efficiency in predicting survival at 12, 24, and 36 months.

As an important component of tumor microenvironment, inflammation plays a complex role in the occurrence and development of cancer; it is considered the seventh hallmark of cancer [4, 8]. In OCRC, due to colorectal obstruction, a large number of bacteria in the intestinal tract proliferated, resulting in dysbacteriosis. Patients usually have more serious local and systemic inflammatory reactions, and even septic shock [9, 26]. Inflammation caused by obstruction leads to aggregation of neutrophils and monocytes, further promoting the development of cancer through the production and release of a large number of inflammatory mediators, transformation factors, cytokines, and chemokines [4, 8]. On the contrary, the local immune response caused by inflammation leads to the increase in monocytes and lymphocytes, which can play as a certain anticancer activity. However, a degree of immunosuppression may also be present in OCRC patients; thus, tumor–host immunity and inflammatory response exhibit a complicated interaction [6, 27, 28]. CRC patients present a heterogeneous immune landscape according to microsatellite status and other factors. Most patients have microsatellite-stable (MSS) tumors with poor immune cell infiltration, and the difference in immune response has a more profound impact on the prognosis of patients with obstruction [29]. The association between MSI status and OCRC deserves further study in the future. Therefore, the changes in immune and inflammatory indicators, such as NLR and LMR, can undoubtedly provide a powerful reference for assessing the prognosis of OCRC, and some studies have also investigated the association between the biomarkers and prognosis of OCRC [23–25].

The association between nutritional indicators and OCRC prognosis deserves attention. The nutritional status of CRC patients, especially those complicated with obstruction, has received increased attention in the first-line clinical practice, and nutritional therapy plays a key role in comprehensive treatment [30, 31]. The nutritional status of patients is generally worse due to the presence of digestive tract obstruction symptoms, and their surgical tolerance, postoperative or post-treatment complication risk, recovery ability, and anti-infection ability are lower than those of patients without obstruction [9, 32]. Therefore, we could better assess the status and prognosis of OCRC patients and make more appropriate therapeutic interventions by focusing on pre-treatment nutritional status. Serum ALB content is a crucial indicator in the clinical front-line of gastrointestinal surgery, which does not only effectively present the nutritional status of patients with obstruction, but also serves as a marker of systemic inflammation; it has been widely included in various scoring systems [7, 16]. Previous study has shown that low CHOL is associated with poor prognosis of CRC [33], and the inclusion of CHOL could also help to evaluate the nutritional status of OCRC patients more effectively.

The conventional NPS contains inflammatory factors such as neutrophils, lymphocytes, monocytes, and nutritional status indicators such as cholesterol and albumin, and has been validated in the prognostic evaluation of CRC [15, 20]. However, OCRC patients would have worse systemic nutritional status and inflammation compared with conventional CRC, and conventional NPS did not have good suitability in OCRC cohort. Therefore, based on the characteristics of OCRC patients with severe inflammatory response and poor immunonutritional status, x-tile software was used to re-intercept the cut-points of NPS factors suitable for this special cohort, and a prognostic scoring system M-NPS suitable for evaluating the inflammatory immunonutritional status of OCRC patients was constructed. OCRC patients had significantly worse prognosis as M-NPS increased, with patients in the M-NPS group 2 having significantly worse OS and DFS than patients in group 0. M-NPS was an important prognostic factor for OCRC patients independent of tumor stage; it can effectively predict the survival of OCRC patients within 36 months. A major advantage of this M-NPS is that it covers clinically focused indicators of inflammation, immunity, and nutritional status in patients with obstruction. The application of scoring can provide reference for OCRC patients to obtain optimal treatment decisions; it could help appropriate patients to obtain more active immunonutritional intervention.

This study has some limitations, mainly in the retrospective analysis and limited sample size. Whether to receive transitional SEMS treatment prior to surgery was not designed separately. Thus, the predictive value of M-NPS should be further evaluated by prospective studies with a large sample size.

## Conclusion

According to the characteristics of OCRC patients, such as severe inflammation and poor nutrition, this study combined the inflammatory, immune, and nutritional indicators to construct a prognostic score, M-NPS, and verified that it is an independent prognostic factor for OS and DFS.

## Data Availability

The datasets analysed during the current study are available from the corresponding author on reasonable request.

## Abbreviations

ASA class: American Society of Anesthesiologists Physical Status Classification
ALB: Albumin
AUC: Area under the curve
BMI: Body Mass Index
CHOL: Total cholesterol
CRC: Colorectal cancer
CT: Computerized tomography
DFS: Disease-free survival
HRs: Hazard ratios
IQR: Interquartile ranges
KM: Kaplan-Meier
LMR: Lymphocyte:monocyte ratio
LNR: Lymph node ratio
mCRC: Metastatic CRC
M-NPS: Modified Naples prognostic score
MRI: Magnetic resonance imaging
NLR: Neutrophil:lymphocyte ratio
NPS: Naples prognostic score
OCRC: Obstructive colorectal cancer
OS: Overall survival
PLR: Platelet:lymphocyte ratio
PNI: Prognostic nutrition index
ROC: Receiver operating characteristic
SEMS: Self-expanding metal stent
SII: Systemic immune–inflammatory index
95%CIs: 95% confidence Intervals
